# Peripheral B Cell Subsets in Autoimmune Diseases: Clinical Implications and Effects of B Cell-Targeted Therapies

**DOI:** 10.1155/2020/9518137

**Published:** 2020-03-23

**Authors:** Wanlin Jin, Zhaohui Luo, Huan Yang

**Affiliations:** Department of Neurology, Xiangya Hospital, Central South University, Changsha, Hunan, China

## Abstract

Antibody-secreting cells (ASCs) play a fundamental role in humoral immunity. The aberrant function of ASCs is related to a number of disease states, including autoimmune diseases and cancer. Recent insights into activated B cell subsets, including naïve B cell to ASC stages and their resultant cellular disturbances, suggest that aberrant ASC differentiation occurs during autoimmune diseases and is closely related to disease severity. However, the mechanisms underlying highly active ASC differentiation and the B cell subsets in autoimmune patients remain undefined. Here, we first review the processes of ASC generation. From the perspective of novel therapeutic target discovery, prediction of disease progression, and current clinical challenges, we further summarize the aberrant activity of B cell subsets including specialized memory CD11c^hi^T-bet^+^ B cells that participate in the maintenance of autoreactive ASC populations. An improved understanding of subgroups may also enhance the knowledge of antigen-specific B cell differentiation. We further discuss the influence of current B cell therapies on B cell subsets, specifically focusing on systemic lupus erythematosus, rheumatoid arthritis, and myasthenia gravis.

## 1. Introduction

Autoreactive antibody-secreting cells (ASCs) refer to short-lived proliferating plasmablasts (PBs) and nonproliferating plasma cells (PCs), with distinct expression profiles, cell morphologies, and a lifespan from B cell lineages [1]. Autoimmune diseases such as systemic lupus erythematosus (SLE) [2], rheumatoid arthritis (RA) [3], and myasthenia gravis (MG) [4] are characterized by T cell hyperactivity and the overproduction of autoantibodies by ASCs, leading to highly activated differentiation to ASCs. For instance, the majority of autoantibodies causing MG are antiacetylcholine receptors (AChR) and AChR^+^CD21^+^ B cells in MG patients positively correlate with anti-AChR antibody production by ASCs in the serum [5], suggesting that hyperactivated antigen-specific B cell differentiation to ASCs represents a precursor of autoreactive ASCs. Other antigen-specific B cells, such as ANA^+^ lgG^+^ switched cells and IgG^+^ PBs, are elevated in SLE and further support the highly connected differentiation to ASCs [4]. In SLE patients, next-generation sequencing (NGS) has shown higher naïve to ASC and IgD^−^ memory to ASC connectivity [6].

This highly activated process of differentiation to ASCs is believed to be induced by the disruption of tolerance checkpoints, which promotes survival of autoreactive ASCs with increasing quantities of autoantibodies [7–9]. Through the detection of B cells that recognize nuclear antigens (ANA^+^ B cells) using flow cytometry, the checkpoints between transitional/naïve and naïve/memory cells have been identified in SLE and healthy individuals but naïve ANA^+^ compartments are defective in SLE [10]. While the numbers of ANA^+^ IgG PCs have been shown to increase, no changes have been found in ANA^+^ transitional, naïve, or switched/unswitched memory B cells in SLE [4], the exact tolerance checkpoints limiting the entrance of autoreactive ASCs are unknown. Challenges in this area include aberrant B cell groups with unknown phenotypes and unknown relationships to ASCs following differentiation in autoimmune diseases. Second, PCs such as pre-PCs, early PCs, short-lived PCs, and long-lived PCs fail to provide precise markers [11], increasing the difficulty in clarifying ASC origin and differentiation. Third, the phenotypes of autoreactive B cells with altered B cell receptor (BCR) repertoires [6, 8] are poorly understood, and pathogenic antibodies generated by different clones of autoreactive B cells may exhibit heterogeneity of effector mechanisms.

Current biological agents targeting B cells including rituximab have been trialed in autoimmune diseases, which to date have shown only limited success, failing to deplete and prevent the replenishment of aberrant ASCs. The reasons for the lack of therapeutic efficacy include memory B cell-mediated relapse [12, 13], some unaffected subsets in peripheral blood [13–17] and in tissue [18, 19], unaffected factors such as BAFF and CD59 [18], and some autoantibody-producing B cell clones protected from rituximab-mediated cytotoxicity [20, 21]. Improving our knowledge of abnormally expanded autoimmune-associated subsets can enhance our understanding of ASC differentiation and explain therapeutic failures. This may reveal more effective targeted therapies and provide potential biomarkers that are appropriate for both diagnostic purposes and prediction of outcome.

We therefore revisited the normal processes of ASCs and conclude possible mechanisms that lead to abnormalities in B cell homeostasis. The existence of specific homing receptors in distinct subpopulations and different activation thresholds amongst the different stages of B cells were used to identify autoimmune-associated subsets [22]. We further summarize the current identified groups and discuss their potential roles as biomarkers for the prediction of organ damage, disease activity, and the influence of current B cell therapy.

## 2. Generalities during ASC Differentiation

### 2.1. Immature B Cells

Under normal conditions, immature B cells are generated in the bone marrow (BM), except for B1 cells that are produced in the fetal liver [23]. Those with autoreactive receptors undergo clonal deletion and sufficient receptor editing to enable effective tolerance [24]. Multireactive BCRs exist when leaving the BM, although they remain unresponsive to antigenic stimulation [25].

### 2.2. Naïve B Cells

Surviving immature/transitional B cells enter the spleen, lymph nodes, or other lymphoid tissues and develop into naïve B cells. Generally, naïve B cells can be divided into B1 cells, marginal zone (MZ) B cells, and follicular (FO) B cells. FO B cells are the most common [26].

### 2.3. Activated B Cells

Activated B cells can differentiate in either a T-independent (TI) or a T-dependent (TD) manner.

In TI responses, all B1 cells, MZ B cells, and FO B cells are activated and differentiate into PBs, although these cells show differential responsiveness to antigens, cytokines, or costimulation [27–29]. FO B cells show limited functionality during ASC differentiation in the absence of T cells compared to B1 and MZ B cells [27]. The reasons for these differences include alterations in TLR amongst the subgroups [28, 30] and low Mzb1 (pERp1) expression of FO B cells [31]. Mzb1 is required for ASC formation in TI [31]. This is highly expressed in B1 and MZ B cells, and its silencing impairs ASC differentiation in TI responses [31]. Since long-lived PCs also exist in T cell-deficient mice after immunization with LPS, TI also induces the formation of PCs [32, 33].

**Figure 1:**
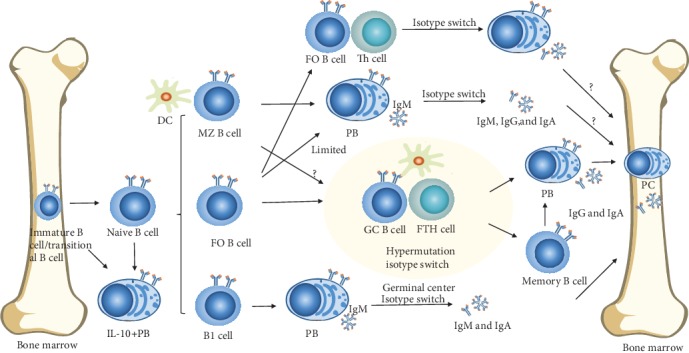
B cells differentiate into ASCs. Differentiation of B cell subsets can be T cell-dependent or T cell-independent. Some PBs develop from immature or naïve B cells and can regulate IL-10 secretion. TD responses include GC reactions and extrafollicular GCs. Activated naïve B cells can develop in a T cell-independent manner while FO B cells have limited functionality. PCs in BM are conventionally derived from PBs produced following GC reactions.

In TD responses, both FO B cells and MZ B cells show functionality [34]. Following activation, both undergo somatic mutations of the variable portion of expressed antibodies to alter and improve antigen specificity and affinity through extrafollicular responses and GC formation [1]. The generated PBs lack the ability to form PCs, and many undergo apoptosis. Extrafollicular growth typically occurs in the medullary cords of lymph nodes and in the T zone-red pulp border of the spleen, with low-levels of hypermutations observed [35]. GC reactions are enhanced by activation-induced cytidine deaminase (AID) [35] and result in the formation of plasma and memory B cells. PCs generated via these methods produce high affinity, class-switched immunoglobulins. Memory B cells can be found in both blood and lymph tissue with lower activation thresholds [36] and rapidly differentiate into ASCs. PCs are home to BMs through CXC-chemokine receptor 4 (CXCR4) and continuously produce antibodies in the absence of antigenic stimulation, providing immediate protection [1]. CD19 niches provide external survival signals and are of great importance to PC survival [37] (Figure 1).

## 3. Potential Mechanism of B Cell Subset Alterations and Failure of Therapy

### 3.1. Mechanism of Self-Tolerance

In BM stage, immature B cells undergo clonal deletion or receptor editing to complete central tolerance, eliminating 20%~50% of self-reactive clones [38]. Additional peripheral tolerance includes anergy that occurs prior to entering the mature naïve B cell compartment [38, 39]. Specifically, BCR, TLR, and cytokines govern both normal and self-reactive antibody responses to antigens [40]. In autoreactive immature and transitional B cells, the BCR/TLR pathway increases AID to establish tolerance [41]. Further differentiation through GC or extrafollicular stimulation is dependent on initial BCR affinity and antigen density [42]. However, the nature of BCR, TLR, and cytokine interactions remains unclear.

### 3.2. Relevant Extrinsic and Intrinsic Factors Sustain Alterations in B Cell Subsets

Autoimmune diseases exhibit abnormal central tolerance with unusual BCRs [43]. Unlike central tolerance in the BM stage, the breakdown of peripheral tolerance can be adverse [24] and additional signals are required to overcome regulatory constraints of peripheral tolerance.

**Figure 2:**
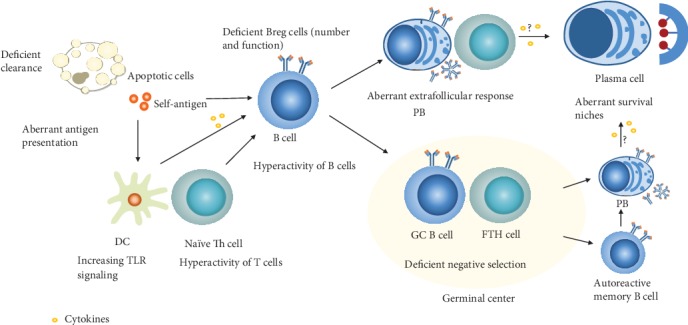
Mechanisms contributing to aberrant ASC secretion. An array of factors regulates the formation of autoreactive ASCs. For example, the deficient clearance of apoptotic material, aberrant antigen presentation, hyperactivated T and B cells, and survival niches for long-lived PC factors with impaired Breg inhibition and aberrant extrafollicular GC formation in disease states.

Extrinsic factors leading to the disruption of B cell tolerance include the deficient clearance of apoptotic cells by macrophages and neutrophils [44], hyperactivity of Th-cells, alterations in dendritic cells [45–48], extrinsic cytokines such as BAFF, IFN^−^*γ*, and IL^−^21 [9, 49–52], TLR stimulation [50, 53, 54], and survival niches for long-lived PCs [37]. Relevant intrinsic factors include changes in major histocompatibility complex (MHC) class II [55], BCR signaling responses [56–58], and TLR responses [59, 60]. These factors in addition to deficient Breg cells [61, 62] and abnormal of extrafollicular germinal centers (GC) formation [6, 63] result in alterations to B cell subsets highly connected to autoreactive ASCs (Figure 2).

### 3.3. Autoreactive PCs and Memory B Cells in Tissues: Difficult Therapeutic Targets

CXCR3, a chemokine receptor, is associated with migration into bone marrow and/or inflamed tissue, and the majority of B cells in healthy individuals lack CXCR3 expression [64]. However, in disease states, PCs are present in the tissue due to inflammatory factors including CXCL10, VCAM-1, and IP-10 [65] and their interaction with CXCR3 [64]. Thymic lymphocytes produce AChR autoantibodies in MG patients either spontaneously or in response to mitogen stimulation [66], suggesting an involvement of autoreactive ASCs in tissues of unknown origin. In addition, TLR4^+^CXCR4^+^PCs undergo significant infiltration into tissues of SLE patients and correlate with the severity of nephritis [67].

AChR-specific CD27^+^ memory B cells are also present in the hyperplastic MG thymus, with unknown specificity [68]. Excluding classical memory B cells, unique peripheral memory cells have been identified in tissues. CD11c^hi^T-bet^+^ B cells are present in nephrotic kidneys, with upregulated chemokine receptors for recruitment to inflamed tissues, such as CCR9 [69]. In addition, circulating CD19^hi^CXCR3^hi^ memory B cells are elevated in SLE and associated with poor clinical outcomes in response to rituximab (RTX) treatment [70], which may also be associated with tissue migration of memory B cells.

## 4. Peripheral B Cell Subset Alterations Associated with ASC Differentiation

Patients with autoimmune diseases show abnormalities during differentiation, with B cell subsets undergoing a wide range of alterations, including transitional B cells, B1, MZ, FO B, and memory B cells giving rise to ASCs, though different stages show preferential responses. In autoimmune diseases, these stages show specific extrinsic and/or intrinsic abnormalities. The clinical significance of these cells is discussed in Table 1.

### 4.1. B1 Cells

B1 cells localizing to the body-cavity serosa either secrete natural antibody spontaneously (B-1a) or respond to TI antigens (B-1a and B-1b) [91]. B1 cells are present in lymphoid organs and blood [91]. Griffin and colleagues defined the phenotypes of circulating human B1 cells as CD20^+^CD27^+^CD43^+^CD70^−^ [92], although this remains controversial.

ASCs from this group play important roles in the production of protective antibodies, serving as major sources of natural IgM [93]. B1 cells can further differentiate into PCs in BM [94]. The pathophysiological functions of B1 cells in human autoimmune diseases require elucidation. Murine studies have proposed that elevated B1 cells are related to defects in macrophage clearance and represent a source of autoantibodies [95]. CD11b^+^ B1 cells increase in the peripheral blood of SLE patients, in which higher CD86 expression is observed, and T cell activity is enhanced [96]. This indicates that B cell subsets are activated and promote immunity. Although Murakami and colleagues reported that the elimination of B-1 cells alleviates clinical responses in autoimmune mice [97], its clinical relevance in patients with autoimmune diseases remains unclear.

### 4.2. Transitional B Cells

Transitional B cells belonging to the immature B2 B cell subset are key players in autoimmune diseases [9]. CD19^+^IgM^hi^IgD^+^CD24^hi^CD38^hi^ transitional B cells are elevated in SLE but are almost absent in healthy controls [6, 9, 72, 98]. Blair and colleagues reported that the majority of CD19^+^CD38^hi^CD24^hi^ B cells are IgM^hi^IgD^hi^CD5^+^CD10^+^CD20^+^CD27^−^CD1d^hi^, which function as regulatory cells [99]. Their regulatory capacity is impaired in SLE [99]. Some B cells using other markers also include transitional B cells [71]. Kosalka and coworkers reported that immature/early^−^transitional B cells (CD27^−^IgD^+^CD21^−^) are elevated [71]. CD21^low^ subsets (immature and activated B cells) are particularly expanded and correlate with lupus nephritis activity [71].

Studies in SLE patients and animal models show that transitional B cells cause the early loss of B-tolerance since a greater percentage of ANA^+^ cells in naïve or new emigrant/transitional B cells are observed in SLE [4, 10, 100].

Cytokines, including BAFF and IFN, mediate transitional B cell abnormalities in SLE. Elevated BAFF expression contributes to transitional B cell expansion [40, 101], and targeting BAFF can recover the normal function of transitional B cells, promoting negative selection of activated autoreactive B cells [10, 102]. IL-6-producing transitional B cells survive in a type I IFN-dependent manner and positively correlate with disease activity in SLE [72]. Dieudonné and colleagues further emphasized the function of IFN by demonstrating that IFN stimulation combined with CD19 downregulation, and impairment of TLR9 responses disturb transitional B cells, resulting in the expansion of ASCs in SLE [9].

### 4.3. MZ B Cells

Following the transition of B cells, some remain in the spleen and develop into MZ B cells. MZ B cells become PBs following antigen presentation and rapidly produce high levels of IgM in a TI manner [103]. MZ B cells are present in human peripheral blood [104]. Although it is unclear whether this population is expanded in patients with autoimmune diseases, MZ B cells contribute to autoreactive clones and their numbers correlate with autoantibody production [105]. They can be rescued by BAFF to undergo expansion [40].

### 4.4. FO B Cells

FO B cells are the largest set of mature B cells following the transitional stage. They circulate freely in the spleen and lymphoid organs, forming an important part of the adaptive immune response, particularly TD responses [26]. TD responses include short-lived PB formation through extrafollicular responses and GC [1].

CD24-activated naïve B cells with a CD19^hi^CD21^−^CD38^low^IgM^low^CD23^−^ phenotype increase in SLE but are absent in healthy controls [6]. These cells exhibit high clonal lineage with ASC populations [6], suggesting that aberrant subsets preferentially undergo differentiation. Increasing ANA^+^ and anti-dsDNA^+^ naïve B cells in SLE patients suggest defective selection at the transitional stage [10], though Suurmond and colleagues reported normal tolerance checkpoints in immature and naïve B cells with increasing total IgG1 PCs [4]. Further studies are now required to explore the exact composition of FO B cells contributing to autoimmune ASC.

### 4.5. Memory B Cells

Memory B cells reflecting autoimmune-associated reactivation are important as these cells possess lower activation thresholds for sustaining autoreactive ASCs with variable responses to therapy. Memory B cells exhibit heterogeneity in SLE, and homogeneous groups are difficult to establish. Usually, immunoglobulin isotopes including IgM, IgD, and CD27 are used to discriminate different memory B cells [106] including CD27^+^IgD^+^, CD27^+^IgD^−^, and CD27^−^IgD^−^ (DN) types. In disease states, different activation markers or clusters of differentiation markers are observed in a range of disease states. Some CD27^−/low^ memory B cells increase in number, including CD11c^hi^T-bet^+^ B cells, CD21^−^B cells, and spleen tyrosine kinase^++^ (Syk) memory-like B cells. The phenotypes defined by CD11c^hi^, CD21, and Syk have overlapping populations, and also overlap with the groups defined by CD27 and IgD [75, 107]. These subsets overlap but are unique. CD11c^hi^ B cells are often characterized by the expression of CD21^−/low^ in disease states [25, 73, 86], while Golinski e al. revealed that less than 10% of CD11c^+^ B cells were CD21^−/low^ in healthy individuals [108]. CD27^−^Syk^++^ memory-like B cells are 64.2 ± 20.9% of CD27^−^CD21^−^ B cells and 67.4 ± 8.0% of CD27^−^IgD^−^CD95^+^ B cells [75].

#### 4.5.1. CD11c^hi^T-bet^+^ B Cells

The number of CD11c^hi^ B cells is related to disease activity, anti-dsDNA levels, and ASC frequency in SLE [73]. Nearly all CD11c^hi^ cells express T-bet in SLE but with lower T-bet expression in healthy individuals [73]. T-bet is required for the generation of CD11c^hi^ cells [109]. *In vitro*, CD11c^hi^ B cells do not spontaneously produce IgG, but are poised to become ASCs and produce the majority of autoantibodies [73]. CD11c^hi^ B cells are CD21^−^CD23^−^ BAFFR (BAFF receptor)^hi^, TACI (transmembrane activator and CAML interactor)^in^, BCMA^lo^ (B cell maturation antigen) and are largely CD27^low^CD38^low^ with switched or unswitched types [73]. The expansion of CD21^−^CD11c^+^ B cells in RA patients supports this finding [86].

CD11c^hi^ B cells possess aberrantly high expression of IL-21R and low expression of CD27 and CD40, which explains their highly activated IL-21 signaling and low threshold for differentiation into ASCs [73]. IL-21 signaling is not unique to SLE naïve B cells and can lead to CD11c expression in healthy donors [73]. CD11c^hi^ B cells not only contribute to the ASCs but also have important function in GC action for antibody-affinity maturation [110].

#### 4.5.2. CD21^-/low^ B Cells

Compared with CD21^+^B cells, CD21^-/low^ B cells have higher numbers of polyreactive clones in both RA patients and healthy donors [25]. In healthy donors, Thorarinsdottir and coworkers reported that circulating CD21^-/low^ B cells were primarily memory B cells and that CD21^-/low^ B cells were less frequent than CD21^+^B cells with 25% CD27^+^ B cells [111]. Lau and colleagues reported that cells in healthy humans undergo active GC reactions with variable gene mutations [112]. Further transcriptional analysis supports the theory that cells are predisposed for differentiation into ASCs after the GC stage, with higher levels of Blimp-1 and T-bet compared with classical memory B cells in healthy donors, especially for CD27^+^ CD21^-/low^ B cells [112].

In disease states, CD21^-/low^ B cells exhibit abnormalities and aberrant expansion in RA [25] and in SLE producing autoantibodies without somatic hypermutation [6] and higher naïve B cell composition [25]. The requirements for differentiation in the pathological state partly result from expanded naïve cell compositions. In RA patients, CD21^-/low^ B cells exhibiting differential responses to BCR, CD40, or TLR9 are significantly expanded; the majority of which express autoreactive antibodies. The cells fail to proliferate or activate through BCR and/or CD40 [25]. At the transcriptional level, B cell activation, trafficking, and proliferation decrease, while the expression of integrins, including *ITGAX*, which encodes CD11c, increases [25].

#### 4.5.3. CD27^+^IgD^+^ B Cells and CD27^+^IgD^−^ B Cells

Several groups have reported that non-witched memory (CD27^+^IgD^+^) B cells decrease in SLE [6, 71, 77, 78, 80, 113] and RA [88], with increases in class-switched memory (CD27^+^IgD^−^) B cells [6, 71, 78–80].

For nonswitched memory (CD27^+^IgD^+^) cells, homogeneous patient cohorts have been assessed in quiescent SLE patients [79]. A decrease in nonswitched memory cells with no increase in other types was observed [79]. The quiescent SLE patients with a low frequency of B cells had lower levels of CD45, which may result from the reduced differentiation to ASCs and tissue homing [113]. IgD^+^CD27^+^IgM^+^ memory B cells have a significantly lower association with disease activity and autoantibody concentrations [77, 78]. The cells overexpress CD95, CD80, CD86, CXCR3, and CXCR4 [77], suggesting they contribute to tissue homing. Rodriguez-Bayona and colleagues reported that the phenotype of IgD^+^CD27^+^IgM^+^ memory B cells was consistent during both active and remission stages [77]. Their origin remains unclear but may arise from B1 and MZ B cells [78, 104].

The numbers of class-switched memory (CD27^+^IgD^−^) B cells increase in SLE [79]. These cells express higher CXCR3 in SLE compared with healthy controls and RA patients, with lower CXCR5 expression [114], which may explain why they are less susceptible to therapy. CXCR5^+^CXCR3^−^ B cells lead to a B cell class switch through the combined stimulation of BCR and TLR [114], suggesting that class-switched memory B cells originate from CXCR5^+^ IgM memory B cells. In *in vitro* studies, lower thresholds were observed due to enhanced CXCR3 expression when stimulated with either CD40L, soluble BAFF, or IL-21, in addition to BCR and IFN^−^*γ* [79, 80].

#### 4.5.4. CD27^−^IgD^−^ B Cells

Double-negative (CD27^−^IgD^−^) memory B cells (DN) represent another class of isotype-switched cells. In healthy individuals, their numbers are small with a higher proportion of IgM memory cells [98], suggesting that they are activated in disease states.

DN cells can be expanded and express somatically mutated VH genes [76]. Their frequency correlates with renal involvement, disease activity, and specific autoantibodies, while RA shows no differences [76]. They lack the expression of FcRH4, with higher mutation rates and recirculation in the peripheral blood compared with CD27^−^ (CD27 negative) memory B cells [76, 77, 98, 115]. This suggests higher activation rates after the GC stage. However, Jacobi et al. observed disease-specific activity and serologic abnormalities with CD27^−^IgD^−^CD95^+^ memory B cell subset, as opposed to CD27^−^IgD^−^ memory B cells [74]. While increasing numbers of double-negative cells are not consistently correlated with anti-dsDNA [74] and the lack of CD27 expression impairs their binding to T cells, their relationship with ASCs requires additional research. DN cells express higher levels of activation markers (CD86, HLA-DR), chemokine receptor CXCR3, and CD71 [74, 113], suggesting an association with aberrant extrafollicular differentiation. Jenks et al. defined CXCR5^−^CD21^−^CD11c^+^ DN cells derived from CXCR5^−^ CD21^−^CD11c^++^IgD^+^CD27^−^ naïve B cells due to additional differentiation to circulating PCs through the extrafollicular activation pathway [107].

#### 4.5.5. CD27^−^Syk^++^ Memory-Like B Cells

SYK, a key element of BCR signaling, is critical for B cell antibody TI/TD responses and memory B cell survival [116]. CD27^−^Syk^++^ memory-like B cells are expanded in SLE, with CD19^++^CD20^++^CD38^−^ phenotypes, primarily CD21^−^ [75]. The main difference with DN or CD95^+^ DN B cell subsets is that more than half of CD27^−^Syk^++^ B cells express IgD [75]. In *in vitro* studies, these cells are produced via stimulation with interferon-*γ*, lipopolysaccharides, or tumor necrosis factor *α*, showing elevated p-Syk expression and differentiation into CD27^++^ IgG secreting cells [75]. Thus, these cells represent the precursors of autoimmune ASC in SLE [75].

### 4.6. ASCs

Autoreactive ASCs produce autoantibodies and can be used to predict disease progression. The increasing number of ASCs not only is responsible for pathogenic autoantibody production but also is associated with accelerated autoimmune disorders [117]. Long-lived PCs in the absence of antigen stimulation represent autoreactive immunological memory cells that secrete pathogenic autoantibodies, but direct studies of autoreactive PCs in humans are challenging since they represent rare and inaccessible cells. The exact origin and pathways of differentiation remain difficult to establish since the markers are unclear [11]. Although it is generally considered that autoreactive ASCs originate from TD responses, elevated MZB1 levels in SLE suggest the contribution of TI responses [118].

In the MuSK MG group, Stathopoulos and coworkers reported that autoreactive ASCs produce MuSK antibodies during relapse [90]. In AChR MG patients, CD19^−^CD138^+^ ASCs significantly increase and strongly correlate with follicular helper T cell frequency in MG patients, while the frequency of follicular helper T cells (FTh) was associated with disease activity [85]. Patients of generalized MG have a higher proportion of CD19^−^CD138^+^ ASCs than clinical forms of ocular MG [85]. In addition, IL-6 and IL-21 which are important to GC activity [119] are increased in the serum [85]. Blocking of IL-21 signaling decreases antibody production [85], suggesting that the function of FTh is in aiding B cell differentiation to CD19^−^CD138^+^ ASCs in an IL-21-dependent manner.

In SLE, ASCs were expanded in the peripheral blood [6, 120]. Using single-cell analysis, ASCs display lower frequencies of SHM and higher mutation frequencies in hypervariable CDR [6]. IgM^+^ ASCs are elevated in SLE patients; a small proportion of which are derived from newly activated naïve B cells [6], suggesting the importance of antigen-driven selection when differentiating towards ASCs and the importance of precursor identification.

#### 4.6.1. TLR4^+^CXCR4^+^ PCs

TLR4^+^CXCR4^+^ PCs are expanded in the blood and renal tissue of SLE [67]. Their non-Ki-67 expression suggests they are nondividing cells and that their frequency correlates with disease activity and renal damage in SLE [67]. In *in vitro* studies, TLR4 inhibitors cause decreased anti-dsDNA IgG secretion [67], suggesting the importance of TLR4 signaling and their contribution to autoantibody production.

#### 4.6.2. HLA-DR^hi^CD27^hi^ PBs

Circulating HLA-DR^hi^CD27^hi^ PBs are elevated in SLE [81]. Compared with CD27^++^CD20^−^CD19^dim^ cells, HLA-DR^hi^CD27^hi^ PBs show a closer correlation with lupus and anti-dsDNA levels [81]. HLA-DR^hi^ PBs are also present in BM and contribute to PC formation in disease states [81]. Further analysis of the chemokines and relevant survival molecules is now required.

#### 4.6.3. RP105^−^ B Cells

RP105 is a B cell surface molecule of TLRs associated with B cell proliferation and death [121]. Korganow and colleagues reported that RP105^−^CD86^+^ and RP105^−^CD38^+^ cells are persistently elevated in SLE, even in quiescent phases [113]. The frequency of RP105^−^ B cells correlates with disease activity in SLE [84]. RP105^−^ B cells cannot be divided into classically categorized B cells and belong to neither GC B cells nor memory B cells but display a highly activated CD95^+^CD86^+^CD38^+^IgD^−^IgM^lo^ phenotype [83]. *In vitro* studies in which autoantibodies and polyclonal immunoglobulins have been produced, implying the influence of ASC composition [83, 84].

**Figure 3:**
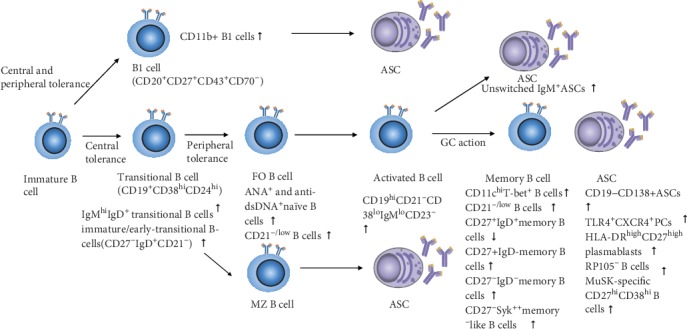
Differentiation of aberrant ASCs and the involved subgroups. From immature B cells to ASCs, B cell subgroups show expressional changes in autoimmune diseases. Unique autoimmune-memory phenotypes include CD11c^hi^ B cells.

Compared with healthy individuals, RP105^−^ B cells demonstrate increasingly higher relative ratios of BCMA/BAFF-R expression in SLE [82], suggesting that RP105^−^ B cells are dependent on BAFF/APRIL when differentiating into ASCs. The exact mechanisms of these pathways now require further elucidation (Figure 3).

## 5. Influence of B Cell Targeting Therapy

Currently, belimumab therapy for SLE and rituximab for RA are approved by the US Food and Drug Administration [122, 123]. Rituximab is recommended for clinical use for severe or MuSK MG [124, 125] and SLE in refractory- and corticosteroid-dependent forms of kidney or central nervous system involvement or severe autoimmune thrombocytopenia [126]. Here, we discuss their effects on different B cell subgroups and their association with clinical efficacy (Table 2).

### 5.1. Anti-CD20 Monoclonal Antibodies

CD20 is a 33 kD protein expressed by all mature B cells, except for plasma B cells. Rituximab (RTX), ofatumumab, and ocrelizumab are monoclonal therapeutic anti-CD20 antibodies considered treatments for autoimmune diseases [127].

RTX is recommended for patients who are refractory to standard therapy in RA [128], severe or MuSK MG [124, 125], and SLE [126]. The depletion of precursor cells that differentiate into autoimmune ASCs is considered the cause of effective treatment [129]. Except for the effect on antibody secretion, RTX mediates trogocytosis of human B cells by producing and releasing IL-6 *in vitro* and has no effect on tumor necrosis factor *α*, IL-1*β*, interferon-*γ*, or IL-10 [130]. In RA, patients have higher levels of IL-6 after 6 months of therapy and high IL-6 levels are also good predictors for RTX response [131, 132]. Third, the few remaining and/or regenerating B cells exhibit incomplete deficiency in costimulatory molecule expression, thus having impaired antigen presentation function [133, 134].

Following their depletion, immature and transitional B cells can be detected for several months [135] and B cell reconstitution is observed. The efficacy of rituximab in autoimmune diseases is mediated by decreased rates of PC synthesis and improved selection for autoreactivity by receptor revision. Numbers of PBs and PCs decrease indirectly after one year of RTX therapy in SLE [136] and 16 months of RTX therapy in RA [137]. Good and shorter clinical responses after RTX therapy are associated with a sustained decrease in anti-dsDNA antibodies for SLE [21, 138, 139] and in anti-CCP autoantibodies for RA [131, 140, 141]. However, the clinical efficacy of RTX varies amongst individual patients. Lacking CD20 expression [142], stem cells, PCs, and PBs are unaffected in some studies [143, 144], and higher PB counts are associated with relapse in 26 weeks in SLE [145]. In tissues of RA patients, unaffected long-lived PCs are also found [18]. In SLE, some patients with peripheral ASCs suppressed have a continuously high anti-dsDNA titer, suggesting the presence of autoantibody-producing long-lived PCs [145]. In addition, a high frequency of memory B cells is also associated with poor clinical responses to RTX [13]. Muto and colleagues have reported that the repopulation of IgD^−^CD27^−^ and IgD^−^CD27^+^ memory B cells is associated with disease activity during relapse after anti-CD20 treatment [12]. Lazarus and colleagues suggested using therapies other than RTX in SLE patients with high levels of IgD^−^CD27^−^ memory B cells and low anti-dsDNA antibody levels [138]. RTX alone may not be sufficient to delete autoreactive clone.

### 5.2. Anti-CD22 Monoclonal Antibodies

CD22 is a transmembrane protein that regulates adhesion and inhibits BCR signaling [146]. CD 22 is expressed on the majority of developing B cells except for plasmablasts and plasma cells [147].

Epratuzumab is a humanized antibody directed against CD22 [148], inhibiting B cell proliferation and maturation and reducing production of proinflammatory cytokines including IL-6 and TNF-*α* [16, 147, 149]. Although phase IIb studies have shown improvements [150], recent phase III data reveal no differences compared with standard therapy without epratuzumab in SLE [151, 152]. Post hoc analysis of open trials of SLE patients with primary SS demonstrates that anti-SSA levels were consistently reduced after epratuzumab treatment [153]. Additional research is required to fully explore responsive clinical subgroups and relevant mechanisms.

Immature B cells, transitional B cells, naïve B cells, and limited memory B cells are affected [16, 154]. CD27^+^ memory B cells are less affected, partly due to low CD22 expression [16] and lower binding with epratuzumab [147]. In an *in vitro* study, epratuzumab binding leads to the expression of CD62L, decreased *β*7 integrin, and increased *β*1 integrin, and the primary effect is observed on CD27^−^ B cells [147]. The unaffected CD27^+^ memory B cells may contribute to the failure of the therapy.

### 5.3. Targeting ASCs with Proteasome Inhibitors

CD19^+^ ASCs in the BM or spleen and CD19^−^ BM ASCs in BM have a similar capacity to contribute to immunological memory [155]. Both contribute to the failure of current therapeutic approaches.

Bortezomib, a proteasome inhibitor, has been used in the treatment of autoantibody-mediated autoimmunity, including SLE [156–158], RA [159], and MuSK MG [160]. Further studies are required to ensure safety, since murine studies showed higher mortality rates after drug use [161]. The drug effectively depletes both short-lived and long-lived PCs in the peripheral blood and bone marrow by ∼50% including the CD19^−^ phenotype by inducing apoptosis [17] with decreasing levels of pathogenic autoantibodies [156]. In addition, fluctuations in anti-dsDNA antibody levels have been observed in relation to each bortezomib cycle [17], suggesting the dynamic depletion of autoreactive ASCs. However, precursor B cells remain unaffected, resulting in the rapid repopulation of ASCs in the absence of bortezomib [17]. Bortezomib withdrawal is accompanied by rapid repopulation of short-lived PCs with increasing autoantibody levels [17].

### 5.4. Targeting B Cell Survival Factors

Blocking targets include BAFF, its homolog APRIL, and their receptors including BAFF-R, BCMA, and TACI. BAFF-R is expressed on the surface of human peripheral B cell subsets excluding PCs and centroblasts located in the dark zone of GCs [162] while BCMA is expressed constitutively by long-lived plasma cells and is important for their survival [163]. TACI is expressed on activated B cells, MZ B cells, switched memory B cells, and PCs [164]. BAFF-R is the major receptor molecule for BAFF-dependent response in the peripheral blood [165], and the interaction of BAFF and BAFF-R is required for the survival and late transition of MZ and mature naïve B cells [105]. Relevant agents include belimumab, tabalumab, atacicept, and blisibimod, which primarily block BAFF.

Belimumab, an inhibitor of BAFF, is recommended for those with active or flaring extrarenal disease in SLE [126]. Belimumab functions rapidly in the early developmental stages of B cells, especially naïve B cells and transitional stages [14]. Malkiel and colleagues elaborated that belimumab treatment restored the censoring of ANA^+^ transitional B cells through anergy [10]. In SLE, earlier longitudinal studies demonstrated that inhibiting BAFF using belimumab selectively reduced the total number of transitional and naïve B cells with no effects on memory B cells [15]. CD11c^+^CD21^−^ B cells [14] and double-negative memory B cells [13, 14] continuously declined with stable PBs [14, 15] and switched memory B cells [13, 14], suggesting the importance of unique memory B cells. PBs slowly decrease after 532 days [13] or 18 months [14]. The level of anti-dsDNA autoantibodies also decreased but only at an early stage [14]. Switch and nonswitch memory B cells and PCs exhibit resistance to belimumab therapy. While the lack of BAFF-R on PCs may be one reason, it cannot be explained why switched memory B cells can survive. These delayed clinical effects require a longer therapeutic regimen.

## 6. Conclusions and Future Perspectives

Autoimmune diseases results from B cell hyperactivity and a disturbance of ASC homeostasis. The development of multichromatic flow cytometry has improved the identification of aberrant B cells and promotes the expression of extrinsic and/or intrinsic molecules. In this review, we have discussed the normal progression towards ASCs (Figure 1), the potential mechanisms of imbalance (Figure 2), and potential B cell subgroups that mediate autoimmune diseases (Figure 3). Aberrantly activated B cells may further contribute to ASCs and their extrinsic and/or intrinsic alterations (Table 1).

The limited success of current B cell therapies coupled with the depletion of precursor B cells is key to the identification of phenotypes of these heterogeneous pathological groups. Technically, more effective identification methods are required. Recombinant antibodies and mass cytometry may aid the discrimination of subgroups.

Moreover, the relationship with autoreactive ASCs, their differentiation, and their sensitivity to chemokine and homing molecules requires further understanding for the generation of long-lived PCs in tissues. Single-cell RNA sequencing and serum proteomics to identify autoantibodies can provide new insight into autoreactive ASC differentiation and identify the landscape of B cells in autoimmune diseases, including additional peripheral tolerance checkpoints, distinct distributions, gene expression analysis, the association amongst subsets, and their underlying mechanisms of differentiation.

In addition, the assessment of self-antigen reactivity of the expanded groups coupled with the analysis of the differential response to therapy may provide more effective targets for refractory groups.

## Figures and Tables

**Table 1:** Summary of circulating aberrant B-subsets corrected with clinical significance in autoimmune diseases.

Disease	B subset	Stage	Extrinsic and/or intrinsic mechanism	Relevance to the diseases	References
SLE	CD21^low^ subsets ↑	Immature and activated B cells		Correlates with lupus nephritis activity	[71]
SLE	IL-6-producing transitional B cells ↑	Transitional B cells	Type I IFN overactivation with NF-*κ*B activation and reduced Bax	Correlates with disease severity	[72]
SLE	CD19^hi^CD21^−^CD38^low^IgM^low^CD23^−^ B cells ↑	Activated naïve B cells		Possible precursors of plasma cells	[6]
SLE	CD23^−^IgD^+^ CD27^−^ activated naïve cells ↑	Activated naïve B cells		Correlates with disease severity	[6]
SLE	CD19^hi^CXCR3^hi^ B cells ↑	Naïve B cells, memory B cells, ASCs	High basal levels of phosphorylated (spleen tyrosine kinase) Syk and ERK1/2 CXCR3 may mediate migration to the sites of inflammation	Poor clinical outcomes following RTX treatment	[65, 70]
SLE	CD11c^hi^ B cells ↑	Unique memory B cells	Lower CD40 and CD27 expression; increased IL-21R expression; activates IL-21 signaling and drives differentiation	Differentiates into autoreactive plasma cells; correlates with disease severity; negatively associated with C3 and C4; can migrate to target tissue	[73]
SLE	TLR-9 expressing B cells ↑	Memory and plasma B cells	Activated TLR-9 signaling	Correlates with anti-dsDNA antibodies.	[59]
SLE	CD27^−^IgD^−^CD95^+^ memory B cells ↑	Memory B cells	Higher levels of CD86, CXCR3, HLA–DR, and CD71	Correlates with disease severity and serological abnormalities	[74]
SLE	CD27^−^ memory like B cells with high SYK ↑	Memory B cells	High expression of p-SYK; enhanced differentiation into CD27^++^ IgG^−^secreting cells; somatically mutated BCR	Correlates with disease severity; candidate source of plasma cells	[75]
SLE	IgD^−^CD27^−^ memory B cells ↑	Memory B cells	Hypermutation in rearranged VH Abs	Correlates with disease severity, active renal disease, and autoantibodies	[76, 77]
SLE	IgD^+^CD27^+^IgM^+^ memory B cells ↓	Memory B cells	BCR signaling abnormalities with Syk and Btk activation	Correlates with disease severity and autoantibodies	[77, 78]
SLE	IgD^−^CD27^+^ memory B cells ↑	Memory B cells	Higher CXCR3 and lower CXCR5 expression; Syk, Btk, and JAK	Less susceptible to therapy	[79, 80]
SLE	TLR4^+^ CXCR4^+^ CD27^hi^CD38^hi^CD138^+^B cells ↑	Plasma cells	TLR4 promotes the secretion of anti-dsDNA IgG	Correlates with disease activity and severe renal damage	[67]
SLE	HLA-DR^hi^CD27^hi^ plasmablasts ↑	Plasmablasts		Elevated levels are associated with lupus and anti-dsDNA	[81]
SLE	RP105^−^B cells ↑	ASC composition	Preferentially expresses BCMA	Elevated levels associated with disease severity	[82–84]
SLE	ARID3a^+^B cells ↑	All stages except for early naïve B cells	Secretes IFN*α*; not mediated by antibody secretion		[85]
RA	CD21^-/low^ B cell ↑	Naïve and memory B cells	Increases B cell activation	Correlates with lymph proliferation	[25, 86]
RA	CD86^+^ B cells ↑	Activated B cells	Possible association with ICOS+ Tfh cells and serum IL-21	Elevated levels associated with disease severity	[87]
RA	IgD^−^CD27^+^ memory B cells ↑	Memory B cells			[88]
RA	IgD^+^CD27^+^ memory B cells ↓	Memory B cells	Impaired IgM-production capacity and altered BCR repertoire	Correlates with disease activity and the anticyclic citrullinated protein antibodies	[88, 89]
MG	MuSK-specific CD27^hi^CD38^hi^ B cells ↑	Autoreactive ASCs		Present during relapse but not remission	[90]
MG	AChR^+^CD21^+^ B cells ↑	Precursors of ASCs?		Elevated levels associated with disease; correlates and anti-AChR antibodies	[5]
MG	CD19^−^CD138^−^ASCs ↑	Plasmablasts	May associate with follicular helper T cells and IL-21	Elevated levels associated with disease severity	[85]

SLE: systemic lupus erythematosus; RA: rheumatoid arthritis; MG: myasthenia gravis.

**Table 2:** Summary of the effects of B cell targeting therapies on circulating B cell subsets.

B cell targeting therapy	Mechanism of action	B subsets affected	B subsets not or less affected	Relevance to clinical relapse	References
Targeting B cell surface antigens					
Anti-CD20	General B cell depletion mediates complement-dependent cytotoxicity	All mature B cells except plasma B cells	Stem cells, PCs, and PBs	IgD^−^CD27^−^ and IgD^−^CD27^+^ memory B cells	[12, 138]
Anti-CD22	Moderate B cell depletion impairs B cell signaling	CD27^−^ transitional and naïve B cells	CD27^+^ memory B cells		[16, 147, 149, 154]
Targeting ASCs with proteasome inhibitors					
Bortezomib	ASC depletion inducts proapoptotic unfolded protein response components and inhibits of NF-*κ*B signaling	Short-lived and long-lived PCs	Precursor B cells	Precursor B cells	[17]
Targeting B cell survival factors					
Belimumab	Impaired B cell survival promotes negative selection of activated autoreactive B cells	Transitional naïve B cells, CD11c^+^CD21^−^ B cells, and double-negative memory B cells	PBs and switched memory B cells		[13–15, 102, 166]

## References

[1] Nutt S. L., Hodgkin P. D., Tarlinton D. M., Corcoran L. M. (2015). The generation of antibody-secreting plasma cells. *Nature Reviews Immunology*.

[2] Suurmond J., Diamond B. (2015). Autoantibodies in systemic autoimmune diseases: specificity and pathogenicity. *Journal of Clinical Investigation*.

[3] Smolen J. S., Aletaha D., Barton A. (2018). Rheumatoid arthritis. *Nature Reviews Disease Primers*.

[4] Suurmond J., Atisha-Fregoso Y., Marasco E. (2019). Loss of an IgG plasma cell checkpoint in patients with lupus. *Journal of Allergy and Clinical Immunology*.

[5] Yin W., Allman W., Ouyang S. (2013). The increased expression of CD21 on AchR specified B cells in patients with myasthenia gravis. *Journal of Neuroimmunology*.

[6] Tipton C. M., Fucile C. F., Darce J. (2015). Diversity, cellular origin and autoreactivity of antibody-secreting cell population expansions in acute systemic lupus erythematosus. *Nature Immunology*.

[7] Meffre E., O'Connor K. C. (2019). Impaired B-cell tolerance checkpoints promote the development of autoimmune diseases and pathogenic autoantibodies. *Immunological Reviews*.

[8] Vander Heiden J. A., Stathopoulos P., Zhou J. Q. (2017). Dysregulation of B cell repertoire formation in myasthenia gravis patients revealed through deep sequencing. *Journal of Immunology*.

[9] Dieudonné Y., Gies V., Guffroy A. (2019). Transitional B cells in quiescent SLE: an early checkpoint imprinted by IFN. *Journal of Autoimmunity*.

[10] Malkiel S., Jeganathan V., Wolfson S. (2016). Checkpoints for autoreactive B cells in the peripheral blood of lupus patients assessed by flow cytometry. *Arthritis & Rheumatology*.

[11] Malkiel S., Barlev A. N., Atisha-Fregoso Y., Suurmond J., Diamond B. (2018). Plasma cell differentiation pathways in systemic lupus erythematosus. *Frontiers in Immunology*.

[12] Muto K., Matsui N., Unai Y. (2017). Memory B cell resurgence requires repeated rituximab in myasthenia gravis. *Neuromuscular Disorders*.

[13] Reddy V., Klein C., Isenberg D. A. (2017). Obinutuzumab induces superior B-cell cytotoxicity to rituximab in rheumatoid arthritis and systemic lupus erythematosus patient samples. *Rheumatology*.

[14] Ramsköld D., Parodis I., Lakshmikanth T. (2019). B cell alterations during BAFF inhibition with belimumab in SLE. *eBioMedicine*.

[15] Jacobi A. M., Huang W., Wang T. (2010). Effect of long-term belimumab treatment on B cells in systemic lupus erythematosus: extension of a phase II, double-blind, placebo-controlled, dose-ranging study. *Arthritis and Rheumatism*.

[16] Jacobi A. M., Goldenberg D. M., Hiepe F., Radbruch A., Burmester G. R., Dörner T. (2008). Differential effects of epratuzumab on peripheral blood B cells of patients with systemic lupus erythematosus versus normal controls. *Annals of the Rheumatic Diseases*.

[17] Alexander T., Cheng Q., Klotsche J. (2018). Proteasome inhibition with bortezomib induces a therapeutically relevant depletion of plasma cells in SLE but does not target their precursors. *European Journal of Immunology*.

[18] Ramwadhdoebe T. H., van Baarsen L. G. M., Boumans M. J. H. (2019). Effect of rituximab treatment on T and B cell subsets in lymph node biopsies of patients with rheumatoid arthritis. *Rheumatology*.

[19] Uzzan M., Ko H. M., Rosenstein A. K., Pourmand K., Colombel J. F., Mehandru S. (2018). Efficient long-term depletion of CD20^+^ B cells by rituximab does not affect gut-resident plasma cells. *Annals of the New York Academy of Sciences*.

[20] Gong Q., Ou Q., Ye S. (2005). Importance of cellular microenvironment and circulatory dynamics in B cell immunotherapy. *Journal of Immunology*.

[21] Cambridge G., Leandro M. J., Teodorescu M. (2006). B cell depletion therapy in systemic lupus erythematosus: effect on autoantibody and antimicrobial antibody profiles. *Arthritis and Rheumatism*.

[22] Tangye S. G., Avery D. T., Deenick E. K., Hodgkin P. D. (2003). Intrinsic differences in the proliferation of naive and memory human B cells as a mechanism for enhanced secondary immune responses. *Journal of Immunology*.

[23] Montecino-Rodriguez E., Dorshkind K. (2012). B-1 B cell development in the fetus and adult. *Immunity*.

[24] Nemazee D. (2017). Mechanisms of central tolerance for B cells. *Nature Reviews Immunology*.

[25] Isnardi I., Ng Y. S., Menard L. (2010). Complement receptor 2/CD21- human naive B cells contain mostly autoreactive unresponsive clones. *Blood*.

[26] Allman D., Pillai S. (2008). Peripheral B cell subsets. *Current Opinion in Immunology*.

[27] Genestier L., Taillardet M., Mondiere P., Gheit H., Bella C., Defrance T. (2007). TLR agonists selectively promote terminal plasma cell differentiation of B cell subsets specialized in thymus-independent responses. *Journal of Immunology*.

[28] Meyer-Bahlburg A., Rawlings D. J. (2012). Differential impact of Toll-like receptor signaling on distinct B cell subpopulations. *Frontiers in Bioscience*.

[29] Kaminski D. A., Stavnezer J. (2006). Enhanced IgA class switching in marginal zone and B1 B cells relative to follicular/B2 B cells. *Journal of Immunology*.

[30] Ma K., Li J., Fang Y., Lu L. (2015). Roles of B cell-intrinsic TLR signals in systemic lupus erythematosus. *International Journal of Molecular Sciences*.

[31] Andreani V., Ramamoorthy S., Pandey A. (2018). Cochaperone Mzb1 is a key effector of Blimp1 in plasma cell differentiation and *β*1-integrin function. *Proceedings of the National Academy of Sciences of the United States of America*.

[32] Bortnick A., Chernova I., Quinn W. J., Mugnier M., Cancro M. P., Allman D. (2012). Long-lived bone marrow plasma cells are induced early in response to T cell-independent or T cell-dependent antigens. *The Journal of Immunology*.

[33] Racine R., McLaughlin M., Jones D. D. (2011). IgM production by bone marrow plasmablasts contributes to long-term protection against intracellular bacterial infection. *The Journal of Immunology*.

[34] Arnon T. I., Horton R. M., Grigorova I. L., Cyster J. G. (2013). Visualization of splenic marginal zone B-cell shuttling and follicular B-cell egress. *Nature*.

[35] MacLennan I. C. M., Toellner K.-M., Cunningham A. F. (2003). Extrafollicular antibody responses. *Immunological Reviews*.

[36] Phan T. G., Tangye S. G. (2017). Memory B cells: total recall. *Current Opinion in Immunology*.

[37] Radbruch A., Muehlinghaus G., Luger E. O. (2006). Competence and competition: the challenge of becoming a long-lived plasma cell. *Nature Reviews Immunology*.

[38] Hoffman W., Lakkis F. G., Chalasani G. (2016). B cells, antibodies, and more. *Clinical Journal of the American Society of Nephrology*.

[39] Wardemann H., Yurasov S., Schaefer A., Young J. W., Meffre E., Nussenzweig M. C. (2003). Predominant autoantibody production by early human B cell precursors. *Science*.

[40] Sindhava V. J., Oropallo M. A., Moody K. (2017). A TLR9-dependent checkpoint governs B cell responses to DNA-containing antigens. *Journal of Clinical Investigation*.

[41] Kuraoka M., Snowden P. B., Nojima T. (2017). BCR and endosomal TLR signals synergize to increase AID expression and establish central B cell tolerance. *Cell Reports*.

[42] Paus D., Phan T. G., Chan T. D., Gardam S., Basten A., Brink R. (2006). Antigen recognition strength regulates the choice between extrafollicular plasma cell and germinal center B cell differentiation. *Journal of Experimental Medicine*.

[43] Samuels J., Ng Y. S., Coupillaud C., Paget D., Meffre E. (2005). Impaired early B cell tolerance in patients with rheumatoid arthritis. *Journal of Experimental Medicine*.

[44] Abdolmaleki F., Farahani N., Gheibi Hayat S. M. (2018). The role of efferocytosis in autoimmune diseases. *Frontiers in Immunology*.

[45] Chung C. Y. J., Ysebaert D., Berneman Z. N., Cools N. (2013). Dendritic cells: cellular mediators for immunological tolerance. *Clinical and Developmental Immunology*.

[46] Ganguly D., Haak S., Sisirak V., Reizis B. (2013). The role of dendritic cells in autoimmunity. *Nature Reviews Immunology*.

[47] Deane J. A., Pisitkun P., Barrett R. S. (2007). Control of toll-like receptor 7 expression is essential to restrict autoimmunity and dendritic cell proliferation. *Immunity*.

[48] Das A., Heesters B. A., Bialas A. (2017). Follicular dendritic cell activation by TLR ligands promotes autoreactive B cell responses. *Immunity*.

[49] Lai Y., Dong C. (2016). Therapeutic antibodies that target inflammatory cytokines in autoimmune diseases. *International Immunology*.

[50] Groom J. R., Fletcher C. A., Walters S. N. (2007). BAFF and MyD88 signals promote a lupuslike disease independent of T cells. *Journal of Experimental Medicine*.

[51] Leonard W. J., Wan C. K. (2016). IL-21 Signaling in immunity. *F1000Research*.

[52] Pollard K., Cauvi D., Toomey C., Morris K. V., Kono D. H. (2013). Interferon-*γ* and systemic autoimmunity. *Discovery Medicine*.

[53] Herlands R. A., Christensen S. R., Sweet R. A., Hershberg U., Shlomchik M. J. (2008). T cell-independent and toll-like receptor-dependent antigen-driven activation of autoreactive B cells. *Immunity*.

[54] Lau C. M., Broughton C., Tabor A. S. (2005). RNA-associated autoantigens activate B cells by combined B cell antigen receptor/Toll-like receptor 7 engagement. *Journal of Experimental Medicine*.

[55] Hervé M., Isnardi I., Ng Y. S. (2007). CD40 ligand and MHC class II expression are essential for human peripheral B cell tolerance. *Journal of Experimental Medicine*.

[56] Manjarrez-Orduño N., Marasco E., Chung S. A. (2012). *CSK* regulatory polymorphism is associated with systemic lupus erythematosus and influences B-cell signaling and activation. *Nature Genetics*.

[57] Menard L., Saadoun D., Isnardi I. (2011). The *PTPN22* allele encoding an R620W variant interferes with the removal of developing autoreactive B cells in humans. *Journal of Clinical Investigation*.

[58] Rhee I., Veillette A. (2012). Protein tyrosine phosphatases in lymphocyte activation and autoimmunity. *Nature Immunology*.

[59] Papadimitraki E. D., Choulaki C., Koutala E. (2006). Expansion of toll-like receptor 9-expressing B cells in active systemic lupus erythematosus: implications for the induction and maintenance of the autoimmune process. *Arthritis and Rheumatism*.

[60] Fukui R., Saitoh S.-I., Kanno A. (2011). Unc93B1 restricts systemic lethal inflammation by orchestrating Toll-like receptor 7 and 9 trafficking. *Immunity*.

[61] Oleinika K., Mauri C., Salama A. D. (2019). Effector and regulatory B cells in immune-mediated kidney disease. *Nature Reviews Nephrology*.

[62] Zacca E. R., Onofrio L. I., Acosta C. D. V. (2018). PD-L1^+^ regulatory B cells are significantly decreased in rheumatoid arthritis patients and increase after successful treatment. *Frontiers in Immunology*.

[63] Deng R., Hurtz C., Song Q. (2017). Extrafollicular CD4^+^ T-B interactions are sufficient for inducing autoimmune-like chronic graft-versus-host disease. *Nature Communications*.

[64] Henneken M., Dörner T., Burmester G. R., Berek C. (2005). Differential expression of chemokine receptors on peripheral blood B cells from patients with rheumatoid arthritis and systemic lupus erythematosus. *Arthritis Research & Therapy*.

[65] Xu W., Joo H., Clayton S. (2012). Macrophages induce differentiation of plasma cells through CXCL10/IP-10. *The Journal of Experimental Medicine*.

[66] Levinson A. I., Zweiman B., Lisak R. P., Dziarski A., Moskovitz A. R. (1984). Thymic B-cell activation in myasthenia gravis. *Neurology*.

[67] Ma K., Li J., Wang X. (2018). TLR4^+^CXCR4^+^ plasma cells drive nephritis development in systemic lupus erythematosus. *Annals of the Rheumatic Diseases*.

[68] Yi J. S., Guptill J. T., Stathopoulos P., Nowak R. J., O’Connor K. C. (2018). B cells in the pathophysiology of myasthenia gravis. *Muscle & Nerve*.

[69] Chung A. C. K., Lan H. Y. (2011). Chemokines in renal injury. *Journal of the American Society of Nephrology*.

[70] Nicholas M. W., Dooley M. A., Hogan S. L. (2008). A novel subset of memory B cells is enriched in autoreactivity and correlates with adverse outcomes in SLE. *Clinical Immunology*.

[71] Kosalka J., Jakiela B., Musial J. (2016). Changes of memory B- and T-cell subsets in lupus nephritis patients. *Folia Histochemica et Cytobiologica*.

[72] Liu M., Guo Q., Wu C. (2019). Type I interferons promote the survival and proinflammatory properties of transitional B cells in systemic lupus erythematosus patients. *Cellular & Molecular Immunology*.

[73] Wang S., Wang J., Kumar V. (2018). IL-21 drives expansion and plasma cell differentiation of autoreactive CD11c^hi^T-bet^+^ B cells in SLE. *Nature Communications*.

[74] Jacobi A. M., Reiter K., Mackay M. (2008). Activated memory B cell subsets correlate with disease activity in systemic lupus erythematosus: delineation by expression of CD27, IgD, and CD95. *Arthritis and Rheumatism*.

[75] Fleischer S. J., Giesecke C., Mei H. E., Lipsky P. E., Daridon C., Dörner T. (2014). Increased frequency of a unique spleen tyrosine kinase bright memory B cell population in systemic lupus erythematosus. *Arthritis & Rheumatology*.

[76] Wei C., Anolik J., Cappione A. (2007). A new population of cells lacking expression of CD27 represents a notable component of the B cell memory compartment in systemic lupus erythematosus. *The Journal of Immunology*.

[77] Rodríguez-Bayona B., Ramos-Amaya A., Pérez-Venegas J. J., Rodríguez C., Brieva J. A. (2010). Decreased frequency and activated phenotype of blood CD27 IgD IgM B lymphocytes is a permanent abnormality in systemic lupus erythematosus patients. *Arthritis Research & Therapy*.

[78] Iwata S., Tanaka Y. (2016). B-cell subsets, signaling and their roles in secretion of autoantibodies. *Lupus*.

[79] Odendahl M., Jacobi A., Hansen A. (2000). Disturbed peripheral B lymphocyte homeostasis in systemic lupus erythematosus. *The Journal of Immunology*.

[80] Tanaka Y., Kubo S., Iwata S., Yoshikawa M., Nakayamada S. (2018). B cell phenotypes, signaling and their roles in secretion of antibodies in systemic lupus erythematosus. *Clinical Immunology*.

[81] Jacobi A. M., Mei H., Hoyer B. F. (2009). HLA-DR^high^/CD27^high^ plasmablasts indicate active disease in patients with systemic lupus erythematosus. *Annals of the Rheumatic Diseases*.

[82] Koarada S., Tada Y., Sohma Y. (2010). Autoantibody-producing RP105^–^ B cells, from patients with systemic lupus erythematosus, showed more preferential expression of BCMA compared with BAFF-R than normal subjects. *Rheumatology*.

[83] Koarada S., Tada Y., Suematsu R. (2012). Phenotyping of P105-negative B cell subsets in patients with systemic lupus erythematosus. *Clinical and Developmental Immunology*.

[84] Koarada S., Tada Y., Ushiyama O. (1999). B cells lacking RP105, a novel B cell antigen, in systemic lupus erythematosus. *Arthritis and Rheumatism*.

[85] Zhang C. J., Gong Y., Zhu W. (2016). Augmentation of circulating follicular helper T cells and their impact on autoreactive B cells in myasthenia gravis. *The Journal of Immunology*.

[86] Rubtsov A. V., Rubtsova K., Fischer A. (2011). Toll-like receptor 7 (TLR7)–driven accumulation of a novel CD11c^+^ B-cell population is important for the development of autoimmunity. *Blood*.

[87] Wang J., Shan Y., Jiang Z. (2013). High frequencies of activated B cells and T follicular helper cells are correlated with disease activity in patients with new-onset rheumatoid arthritis. *Clinical and Experimental Immunology*.

[88] Sellam J., Rouanet S., Hendel-Chavez H. (2011). Blood memory B cells are disturbed and predict the response to rituximab in patients with rheumatoid arthritis. *Arthritis and Rheumatism*.

[89] Hu F., Zhang W., Shi L. (2018). Impaired CD27^+^IgD^+^ B cells with altered gene signature in rheumatoid arthritis. *Frontiers in Immunology*.

[90] Stathopoulos P., Kumar A., Nowak R. J., O’Connor K. C. (2017). Autoantibody-producing plasmablasts after B cell depletion identified in muscle-specific kinase myasthenia gravis. *JCI Insight*.

[91] Baumgarth N. (2011). The double life of a B-1 cell: self-reactivity selects for protective effector functions. *Nature Reviews Immunology*.

[92] Griffin D. O., Holodick N. E., Rothstein T. L. (2011). Human B1 cells in umbilical cord and adult peripheral blood express the novel phenotype CD20^+^CD27^+^CD43^+^CD70^−^. *The Journal of Experimental Medicine*.

[93] Kaveri S. V., Silverman G. J., Bayry J. (2012). Natural IgM in immune equilibrium and harnessing their therapeutic potential. *Journal of Immunology*.

[94] Fairfax K. A., Corcoran L. M., Pridans C. (2007). Different kinetics of blimp-1 induction in B cell subsets revealed by reporter gene. *The Journal of Immunology*.

[95] Qian Y., Conway K. L., Lu X., Seitz H. M., Matsushima G. K., Clarke S. H. (2006). Autoreactive MZ and B-1 B-cell activation by *Fas^lpr^* is coincident with an increased frequency of apoptotic lymphocytes and a defect in macrophage clearance. *Blood*.

[96] Griffin D. O., Rothstein T. L. (2011). A small CD11b^+^ human B1 cell subpopulation stimulates T cells and is expanded in lupus. *The Journal of Experimental Medicine*.

[97] Murakami M., Yoshioka H., Shirai T., Tsubata T., Honjo T. (1995). Prevention of autoimmune symptoms in autoimmune-prone mice by elimination of B-1 cells. *International Immunology*.

[98] Wehr C., Eibel H., Masilamani M. (2004). A new CD21^low^ B cell population in the peripheral blood of patients with SLE. *Clinical Immunology*.

[99] Blair P. A., Noreña L. Y., Flores-Borja F. (2010). CD19^+^CD24^hi^CD38^hi^ B Cells Exhibit Regulatory Capacity in Healthy Individuals but Are Functionally Impaired in Systemic Lupus Erythematosus Patients. *Immunity*.

[100] Yurasov S., Wardemann H., Hammersen J. (2005). Defective B cell tolerance checkpoints in systemic lupus erythematosus. *Journal of Experimental Medicine*.

[101] Landolt-Marticorena C., Wither R., Reich H. (2011). Increased expression of B cell activation factor supports the abnormal expansion of transitional B cells in systemic lupus erythematosus. *The Journal of Rheumatology*.

[102] Huang W., Quach T. D., Dascalu C. (2018). Belimumab promotes negative selection of activated autoreactive B cells in systemic lupus erythematosus patients. *JCI Insight*.

[103] Balázs M., Martin F., Zhou T., Kearney J. F. (2002). Blood dendritic cells interact with splenic marginal zone B cells to initiate T-independent immune responses. *Immunity*.

[104] Weller S., Braun M. C., Tan B. K. (2004). Human blood IgM "memory" B cells are circulating splenic marginal zone B cells harboring a prediversified immunoglobulin repertoire. *Blood*.

[105] Oldham A. L., Miner C. A., Wang H. C., Webb C. F. (2011). The transcription factor bright plays a role in marginal zone B lymphocyte development and autoantibody production. *Molecular Immunology*.

[106] Kaminski D. A., Wei C., Qian Y., Rosenberg A. F., Sanz I. (2012). Advances in human B cell phenotypic profiling. *Frontiers in Immunology*.

[107] Jenks S. A., Cashman K. S., Zumaquero E. (2018). Distinct effector B cells induced by unregulated Toll-like receptor 7 contribute to pathogenic responses in systemic lupus erythematosus. *Immunity*.

[108] Golinski M.-L., Demeules M., Derambure C. (2020). CD11c^+^ B cells are mainly memory cells, precursors of antibody secreting cells in healthy donors. *Frontiers in Immunology*.

[109] Naradikian M. S., Myles A., Beiting D. P. (2016). Cutting edge: IL-4, IL-21, and IFN-*γ* interact to govern T-bet and CD11c expression in TLR-activated B cells. *Journal of Immunology*.

[110] Zhang W., Zhang H., Liu S. (2019). Excessive CD11c^+^Tbet^+^ B cells promote aberrant T_FH_ differentiation and affinity-based germinal center selection in lupus. *Proceedings of the National Academy of Sciences of the United States of America*.

[111] Thorarinsdottir K., Camponeschi A., Cavallini N. (2016). CD21^–/low^ B cells in human blood are memory cells. *Clinical and Experimental Immunology*.

[112] Lau D., Lan L. Y. L., Andrews S. F. (2017). Low CD21 expression defines a population of recent germinal center graduates primed for plasma cell differentiation. *Science Immunology*.

[113] Korganow A. S., Knapp A. M., Nehme-Schuster H. (2010). Peripheral B cell abnormalities in patients with systemic lupus erythematosus in quiescent phase: decreased memory B cells and membrane CD19 expression. *Journal of Autoimmunity*.

[114] Nakayamada S., Iwata S., Tanaka Y. (2015). Relevance of lymphocyte subsets to B cell-targeted therapy in systemic lupus erythematosus. *International Journal of Rheumatic Diseases*.

[115] Fecteau J. F., Cote G., Neron S. (2006). A new memory CD27^–^IgG^+^ B cell population in peripheral blood expressing V_H_ genes with low frequency of somatic mutation. *The Journal of Immunology*.

[116] Ackermann J. A., Nys J., Schweighoffer E., McCleary S., Smithers N., Tybulewicz V. L. J. (2015). Syk tyrosine kinase is critical for B cell antibody responses and memory B cell survival. *Journal of Immunology*.

[117] Hoyer B. F., Moser K., Hauser A. E. (2004). Short-lived plasmablasts and long-lived plasma cells contribute to chronic humoral autoimmunity in NZB/W mice. *Journal of Experimental Medicine*.

[118] Miyagawa-Hayashino A., Yoshifuji H., Kitagori K. (2018). Increase of MZB1 in B cells in systemic lupus erythematosus: proteomic analysis of biopsied lymph nodes. *Arthritis Research & Therapy*.

[119] Sweet R. A., Lee S. K., Vinuesa C. G. (2012). Developing connections amongst key cytokines and dysregulated germinal centers in autoimmunity. *Current Opinion in Immunology*.

[120] Espeli M., Bökers S., Giannico G. (2011). Local renal autoantibody production in lupus nephritis. *Journal of the American Society of Nephrology*.

[121] Kimoto M., Nagasawa K., Miyake K. (2003). Role of TLR4/MD-2 and RP105/MD-1 in innate recognition of lipopolysaccharide. *Scandinavian Journal of Infectious Diseases*.

[122] Horowitz D. L., Furie R. (2012). Belimumab is approved by the FDA: what more do we need to know to optimize decision making?. *Current Rheumatology Reports*.

[123] Kesharwani D., Paliwal R., Satapathy T., Das Paul S. (2019). Rheumatiod arthritis: an updated overview of latest therapy and drug delivery. *Journal of Pharmacopuncture*.

[124] Gilhus N. E., Skeie G. O., Romi F., Lazaridis K., Zisimopoulou P., Tzartos S. (2016). Myasthenia gravis — autoantibody characteristics and their implications for therapy. *Nature Reviews Neurology*.

[125] Sanders D. B., Wolfe G. I., Benatar M. (2016). International consensus guidance for management of myasthenia gravis: executive summary. *Neurology*.

[126] Fanouriakis A., Kostopoulou M., Alunno A. (2019). 2019 update of the EULAR recommendations for the management of systemic lupus erythematosus. *Annals of the Rheumatic Diseases*.

[127] Iorio R., Damato V., Alboini P. E., Evoli A. (2015). Efficacy and safety of rituximab for myasthenia gravis: a systematic review and meta-analysis. *Journal of Neurology*.

[128] Smolen J. S., Landewé R., Bijlsma J. (2017). EULAR recommendations for the management of rheumatoid arthritis with synthetic and biological disease-modifying antirheumatic drugs: 2016 update. *Annals of the Rheumatic Diseases*.

[129] Huang H., Benoist C., Mathis D. (2010). Rituximab specifically depletes short-lived autoreactive plasma cells in a mouse model of inflammatory arthritis. *Proceedings of the National Academy of Sciences of the United States of America*.

[130] Jones J. D., Hamilton B. J., Skopelja S., Rigby W. F. C. (2014). Induction of interleukin-6 production by rituximab in human B cells. *Arthritis & Rheumatology*.

[131] Lal P., Su Z., Holweg C. T. J. (2011). Inflammation and autoantibody markers identify rheumatoid arthritis patients with enhanced clinical benefit following rituximab treatment. *Arthritis and Rheumatism*.

[132] Barahona Correa J. E., Franco Cortés M. A., Ángel Uribe J., Rodríguez Camacho L. S. (2018). Comparison of plasma cytokine levels before and after treatment with rituximab in patients with rheumatoid arthritis and systemic lupus erythematosus-associated polyautoimmunity. *Universitas Médica*.

[133] Tokunaga M., Fujii K., Saito K. (2005). Down-regulation of CD40 and CD80 on B cells in patients with life-threatening systemic lupus erythematosus after successful treatment with rituximab. *Rheumatology*.

[134] Liossis S.-N. C., Sfikakis P. P. (2008). Rituximab-induced B cell depletion in autoimmune diseases: potential effects on T cells. *Clinical Immunology*.

[135] Leandro M. J., Cambridge G., Edwards J. C., Ehrenstein M. R., Isenberg D. A. (2005). B-cell depletion in the treatment of patients with systemic lupus erythematosus: a longitudinal analysis of 24 patients. *Rheumatology*.

[136] Anolik J. H., Barnard J., Cappione A. (2004). Rituximab improves peripheral B cell abnormalities in human systemic lupus erythematosus. *Arthritis and Rheumatism*.

[137] Thurlings R. M., Vos K., Wijbrandts C. A., Zwinderman A. H., Gerlag D. M., Tak P. P. (2008). Synovial tissue response to rituximab: mechanism of action and identification of biomarkers of response. *Annals of the Rheumatic Diseases*.

[138] Lazarus M. N., Turner-Stokes T., Chavele K.-M., Isenberg D. A., Ehrenstein M. R. (2012). B-cell numbers and phenotype at clinical relapse following rituximab therapy differ in SLE patients according to anti-dsDNA antibody levels. *Rheumatology*.

[139] Cambridge G., Isenberg D. A., Edwards J. C. W. (2008). B cell depletion therapy in systemic lupus erythaematosus: relationships among serum B lymphocyte stimulator levels, autoantibody profile and clinical response. *Annals of the Rheumatic Diseases*.

[140] Gardette A., Ottaviani S., Tubach F. (2014). High anti-CCP antibody titres predict good response to rituximab in patients with active rheumatoid arthritis. *Joint, Bone, Spine*.

[141] Couderc M., Mathieu S., Pereira B., Glace B., Soubrier M. (2013). Predictive factors of rituximab response in rheumatoid arthritis: results from a French university hospital. *Arthritis Care & Research*.

[142] Edwards J. C. W., Szczepański L., Szechiński J. (2004). Efficacy of B-cell-targeted therapy with rituximab in patients with rheumatoid arthritis. *New England Journal of Medicine*.

[143] Leandro M. J., Cambridge G., Ehrenstein M. R., Edwards J. C. W. (2006). Reconstitution of peripheral blood B cells after depletion with rituximab in patients with rheumatoid arthritis. *Arthritis and Rheumatism*.

[144] Vos K., Thurlings R. M., Wijbrandts C. A., van Schaardenburg D., Gerlag D. M., Tak P. P. (2007). Early effects of rituximab on the synovial cell infiltrate in patients with rheumatoid arthritis. *Arthritis and Rheumatism*.

[145] Vital E. M., Dass S., Buch M. H. (2011). B cell biomarkers of rituximab responses in systemic lupus erythematosus. *Arthritis and Rheumatism*.

[146] Dörner T., Shock A., Goldenberg D. M., Lipsky P. E. (2015). The mechanistic impact of CD22 engagement with epratuzumab on B cell function: implications for the treatment of systemic lupus erythematosus. *Autoimmunity Reviews*.

[147] Daridon C., Blassfeld D., Reiter K. (2010). Epratuzumab targeting of CD22 affects adhesion molecule expression and migration of B-cells in systemic lupus erythematosus. *Arthritis Research & Therapy*.

[148] Carnahan J., Wang P., Kendall R. (2003). Epratuzumab, a humanized monoclonal antibody targeting CD22: characterization of *in vitro* properties. *Clinical Cancer Research*.

[149] Fleischer V., Sieber J., Fleischer S. J. (2015). Epratuzumab inhibits the production of the proinflammatory cytokines IL-6 and TNF-*α*, but not the regulatory cytokine IL-10, by B cells from healthy donors and SLE patients. *Arthritis Research & Therapy*.

[150] Wallace D. J., Kalunian K., Petri M. A. (2014). Efficacy and safety of epratuzumab in patients with moderate/severe active systemic lupus erythematosus: results from EMBLEM, a phase IIb, randomised, double-blind, placebo-controlled, multicentre study. *Annals of the Rheumatic Diseases*.

[151] Clowse M. E. B., Wallace D. J., Furie R. A. (2017). Efficacy and safety of epratuzumab in moderately to severely active systemic lupus erythematosus: results from two phase III randomized, double-blind, placebo-controlled trials. *Arthritis & Rheumatology*.

[152] Onuora S. (2016). Epratuzumab not effective in phase III trials. *Nature Reviews Rheumatology*.

[153] Gottenberg J.-E., Dörner T., Bootsma H. (2018). Efficacy of epratuzumab, an anti-CD22 monoclonal IgG antibody, in systemic lupus erythematosus patients with associated Sjögren's syndrome: post hoc analyses from the EMBODY trials. *Arthritis & Rheumatology*.

[154] Dörner T., Kaufmann J., Wegener W. A., Teoh N., Goldenberg D. M., Burmester G. R. (2006). Initial clinical trial of epratuzumab (humanized anti-CD22 antibody) for immunotherapy of systemic lupus erythematosus. *Arthritis Research & Therapy*.

[155] Groves C. J., Carrell J., Grady R. (2018). CD19-positive antibody-secreting cells provide immune memory. *Blood Advances*.

[156] Alexander T., Sarfert R., Klotsche J. (2015). The proteasome inhibitior bortezomib depletes plasma cells and ameliorates clinical manifestations of refractory systemic lupus erythematosus. *Annals of the Rheumatic Diseases*.

[157] Hiepe F., Hoyer B., Alexander T., Taddeo A., Voll R., Radbruch A. (2011). Therapeutic inhibition of proteasomes in systemic lupus erythematosus. *Journal of Translational Medicine*.

[158] Sjowall C., Hjorth M., Eriksson P. (2017). Successful treatment of refractory systemic lupus erythematosus using proteasome inhibitor bortezomib followed by belimumab: description of two cases. *Lupus*.

[159] Lassoued S., Moyano C., Beldjerd M., Pauly P., Lassoued D., Billey T. (2019). Bortezomib improved the joint manifestations of rheumatoid arthritis in three patients. *Joint, Bone, Spine*.

[160] Schneider-Gold C., Reinacher-Schick A., Ellrichmann G., Gold R. (2017). Bortezomib in severe MuSK-antibody positive myasthenia gravis: first clinical experience. *Therapeutic Advances in Neurological Disorders*.

[161] Ikeda T., Fujii H., Nose M. (2017). Bortezomib treatment induces a higher mortality rate in lupus model mice with a higher disease activity. *Arthritis Research & Therapy*.

[162] Smulski C. R., Eibel H. (2018). BAFF and BAFF-receptor in B cell selection and survival. *Frontiers in Immunology*.

[163] O'Connor B. P., Raman V. S., Erickson L. D. (2004). BCMA is essential for the survival of long-lived bone marrow plasma cells. *The Journal of Experimental Medicine*.

[164] Darce J. R., Arendt B. K., Wu X., Jelinek D. F. (2007). Regulated expression of BAFF-binding receptors during human B cell differentiation. *Journal of Immunology*.

[165] Ng L. G., Sutherland A. P. R., Newton R. (2004). B cell-activating factor belonging to the TNF family (BAFF)-R is the principal BAFF receptor facilitating BAFF costimulation of circulating T and B cells. *Journal of Immunology*.

[166] Stohl W., Merrill J. T., McKay J. D. (2013). Efficacy and safety of belimumab in patients with rheumatoid arthritis: a phase II, randomized, double-blind, placebo-controlled, dose-ranging study. *The Journal of Rheumatology*.

